# The Clinical Concept of Fibromyalgia as a Changing Paradigm in the Past 20 Years

**DOI:** 10.1155/2012/184835

**Published:** 2011-10-29

**Authors:** Mary-Ann Fitzcharles, Muhammad B. Yunus

**Affiliations:** ^1^Division of Rheumatology, McGill University, Montreal, QC, Canada H3G 1A4; ^2^Alan Edwards Pain Management Unit, McGill University Health Center, Montreal, QC, Canada H3G 1A4; ^3^Montreal General Hospital, McGill University Health Centre, Montreal, QC, Canada H3G 1A4; ^4^Section of Rheumatology, Department of Medicine, University of Illinois College of Medicine at Peoria, Peoria, IL, USA

## Abstract

Fibromyalgia (FMS) is a valid clinical condition that affects 2%–4% of the population with a pivot symptom of widespread body pain. The cause and cure of FMS are as yet unknown. The concept of FMS has evolved over the past two decades to incorporate symptoms beyond pain as contributing to the global spectrum of suffering. FMS is now recognized to be grounded in the neurological domain with evidence of dysregulation of pain processing. Appreciation of the neurophysiologic mechanisms operative in FMS has contributed to rational treatment recommendations, although a “gold standard treatment” does not currently exist. Ideal treatments for FMS patients should be individualized with emphasis on active patient participation, good health practices, and multimodal intervention, incorporating nonpharmacologic and pharmacologic treatments. Predictors of outcome, which is favourable in over 50% of patients, are unknown, but those with better outcome do more physical activity and use fewer medications.

## 1. Introduction

### 1.1. The Coming of Age after 20 Years

Fibromyalgia (FMS) is a condition characterized by the pivot symptom of pain throughout the body, and with abnormality centered in the nervous system [1]. Over the past 20 years, knowledge regarding both the clinical as well as the neurophysiological basis for this condition has accumulated. FMS affects 2%–4% of populations worldwide and is a cause of considerable suffering and functional impairment [2]. The clinical concept of FMS was initially described by Yunus and colleagues and crystallized by the publication of the 1990 American College of Rheumatology (ACR) criteria for the classification of FMS [3, 4]. 

An evolution of the clinical understanding of FMS over the last two decades has emphasised the importance of symptoms beyond pain which form an integral part of this condition and contribute to global suffering. In this context, it became necessary for the criteria for a diagnosis of FMS to be reevaluated. The coming of age of FMS was heralded by the publication of updated criteria for the diagnosis of FMS, taking into consideration additional symptoms that are present to a variable degree in individual patients [5]. In addition, the new concept of FMS recognizes that symptoms are not an all-or-none phenonemon, but can be expressed with varying severity with periods of waxing and waning [6].

Neurophysiological studies have contributed to the acceptance of FMS as a valid condition. Demonstration of objective changes in the research setting has given clinicians the confidence to acknowledge a condition that presents with only subjective complaint and no objective clinical findings [1]. Dysregulation of pain processing has been demonstrated at various levels in the nervous system, but we still lack an objective test in the clinical setting to confirm a diagnosis or gauge response to treatments [1, 7]. However, this is no different than other well-recognized conditions, for example, irritable bowel syndrome (IBS), migraine, and depression. It is undeniable that depression is a serious condition and yet it lacks an objective test.

Even with objective scientific support of abnormality, some scepticism still exists regarding the validity of subjective complaints requiring complete reliance on the practice of the art of medicine [8]. The controversy regarding the existence of this syndrome should now be put to rest. Efforts should be directed towards better understanding of the neurophysiological abnormalities, improved clinical recognition of patients, and translation of mechanistic studies into optimizing treatments. In this paper, we will present current concepts of FMS, which can be applied to the rational management of these patients. This paper will address current concepts and challenges pertaining to the clinical understanding of FMS.

## 2. Methods

This paper is based on a review of the literature achieved by a comprehensive literature search using MEDLINE, CINAHL, Cochrane, PUBMED, EMBASE, Cochrane Library, and PsycINFO. MEDLINE is widely used as a premier source for biographic coverage of the literature and the CINAHL for nursing literature. In addition to the formal search, a manual search from the references cited by original studies and reviews was also used where indicated.

### 2.1. The Clinical Challenge Remains

Clinicians are traditionally skeptical of any condition wherein there is disconnection between complaint and physical examination findings. This was first evident with the construct of phantom limb syndrome, now accepted as a real phenomenon and cause of pain in the absence of anatomical tissue in the periphery [9]. Beginning in the 1980's, there were emerging reports that body pain, in the absence of tissue damage, could be present [3, 10, 11]. These patients were mostly referred to rheumatologists in order to rule out some connective tissue disease. The notion that FMS is indeed a true entity follows publication of neurophysiological studies attesting to objective findings in a clinical condition that was often perceived to be nebulous.

Beginning with their landmark study in 1981, Yunus and coworkers have championed the recognition of this condition over the past three decades [3]. In this paper, the authors emphasized that FMS is more than pain, adding many other symptoms or associated conditions, for example, fatigue, stiffness (which is a prominent symptom in some patients), poor sleep, morning fatigue, tension-type headache, migraine, IBS, subjective swelling (which often leads to a misdiagnosis of rheumatoid arthritis), subjective numbness, anxiety, stress and depression, as well as modulating factors of pain and stiffness [3].

There have been physicians who have disputed the validity of a condition presenting with subjective complaints and associated with considerable functional impairment, without objective clinical findings. Some have even suggested that FMS was mostly a manifestation of depression [12, 13]. However, depression and FMS are biologically two different diseases, including an absence of central sensitization in depression by almost all stimuli [14, 15].

The clinical challenge of this condition remains as there is still no objective clinical finding or test to confirm the diagnosis, or gauge severity of symptoms. Physicians are required to assess this syndrome on the basis of subjective report only. This has fostered a sense of clinical uncertainty leading physicians to often consider a diagnosis of FMS only when other possible diagnoses have been excluded [16]. This insecurity by health care professionals may be a factor leading to frequent use of unnecessary investigations and can contribute to excessive medicalization of patients. It has been clearly documented that a definite diagnosis of FMS leads to reduced health care use and also better global patient health [17, 18].

### 2.2. Fibromyalgia Is More Than Just Pain

Initially, FMS was considered to be a condition of pain, and this concept was reinforced by the 1990 ACR criteria which only included features of pain and localized body tenderness [4]. The understanding of FMS today acknowledges that patients with FMS will have a symptom complex characterized by more than just pain with other complaints present with variable intensity [19]. Besides those mentioned above, that is, fatigue, sleep disturbance, cognitive changes, and mood disorder, other symptoms or associated conditions include restless legs syndrome, periodic limb movements in sleep, temporomandibular disorder (TMD), multiple chemical sensitivity, and interstitial cystitis [20, 21]. Each of these symptoms plays a variable role in the presentation of an individual patient and all contribute to a greater or lesser degree towards the overall effect of impaired quality of life and reduced functional activity.

The typical patient is female in her 40's or 50's with a few years of ill-defined musculoskeletal pain [22]. Onset of symptoms is usually gradual, but occasionally there may be a sudden onset following an identifiable event, such as a medical illness, a mentally stressful incident or physical trauma. Only 5–7% of the FMS patients are males. The clinical characteristics of FMS among men are similar to those in women, except that men have fewer symptoms, fewer pain sites, less frequent fatigue and IBS, and fewer tender points [23]. 

Pain is described as being diffuse, deep, and continuous often with periods of exacerbation. Pain symptoms may be modulated by various factors including psychological stress, excessive physical activity, fatigue, or changes in the weather [24]. Some patients also report a superficial burning quality to pain with increased sensitivity to painful stimuli-termed hyperalgesia, and may also have features of allodynia or pain following an innocuous stimulation such as touch [25]. Pain quality or unpleasantness is an equally important component of the pain experience, but is not commonly measured either in clinical practice or even in the study setting of patients with FMS.

Multiple symptoms contribute to the burden of suffering and are increasingly recognized as important from the patient perspective and require attention for achieving optimal patient care [5, 19, 26]. Nonrestorative sleep is associated with widespread pain [27]. Many components of sleep have been measured as abnormal in FMS patients including sleep latency, sleep disturbance, and impaired daytime functioning [28]. Poor quality and duration of sleep has been shown to have a negative impact upon fatigue and affect [29]. Other sleep disorders such as restless leg syndrome or sleep apnoea may also occur in patients with FMS.

FMS patients can experience important cognitive dysfunction, which associates with pain, but not current depression or anxiety, and includes poor working memory, spatial memory alterations, free recall, and verbal fluency [30–32]. Cognitive symptoms are present in FMS even after adjusting data for age, medications, education, and depression [31]. Cognitive changes were however no different when compared to other pain patients, suggesting that pain per se may affect cognition [33]. Although most patients experience associated symptoms in varying degree, it is not required that these be present for a diagnosis of FMS.

Patients with FMS are heterogeneous and attempts have been made to group patients into categories to help direct treatments and predict outcome [25, 34, 35]. Subgrouping of patients with FMS according to psychological distress, depression in particular, has been most commonly reported, but longitudinal studies using subgroups to direct treatment and predict outcome are still required. In a recent analysis of over 3000 patients in various clinic settings in Germany, patients could be subgrouped into 5 categories depending upon pain characteristics and associated comorbidity of depression [25]. It is however still premature to attempt to categorize patients in the clinical setting, other than to pay particular attention to psychological status.

FMS may accompany other medical, neurological, or rheumatologic illnesses as a comorbid condition [36]. Conditions that have been associated with FMS include amongst others various rheumatologic conditions such as systemic lupus erythematosis and rheumatoid arthritis as well as neurologic disorders such as multiple sclerosis and postpolio syndrome [36, 37]. It is important to appreciate that FMS can coexist with these conditions in order to direct treatment appropriately. For example, a continuous complaint of pain due to FMS in a patient with rheumatoid arthritis would be incorrectly treated by increasing treatments with disease modifying agents, rather than addressing the symptoms associated with FMS.

### 2.3. The Conundrum of Criteria for Diagnosis of Fibromyalgia

Criteria for the classification of FMS were established almost two decades ago and take into account only the symptom of pain [4]. Although the cardinal symptom of FMS remains pain, symptoms of fatigue, sleep disturbance, cognitive changes, mood disorder, and other somatic symptoms contribute to the complexity of this syndrome [19].

The original 1990 criteria for classification of FMS pose at least 2 important practical problems [4]. Firstly, they were developed specifically for the purpose of identifying patients for further research in this condition, and secondly, they addressed only the complaint of pain by means of a report of pain and examination of tender points. These criteria were often erroneously used to validate a diagnosis in individual patients in the clinical setting and did not take into account any other concomitant symptoms or severity of symptoms.

According to the 1990 criteria for a classification of FMS, in addition to widespread body pain, tender points were required to be present in at least 11/18 designated areas [4]. Tender points are located at soft tissue sites and reflect a reduction in pain threshold without underlying tissue pathology. Tender points have elicited considerable debate and their true value has been questioned. Tender point examination is a subjective test, open to individual interpretation and reflects an overall reduction in pain threshold, rather than a pathological process at the soft tissue site [38]. They may be present in normal individuals and can increase with age. Reliability is variable, ranging from good to poor [39, 40]. The association of pain report and tender point count is however poorly correlated, suggesting that these measurements represent different parameters of pain experience in FMS [41].

The correct examination method for tender points is also debatable. Methods that have been used include digital palpation, myalgic scoring, or dolorimetry [42]. Although digital examination is the most commonly used method of assessment, it is often not used by physicians caring for FMS patients [5]. There is also report of poor concurrent validity when tender points were examined digitally or by dolorimetry [43]. A person's current psychological state has been shown to influence measurement of tender points suggesting an association with distress rather than an accurate indicator of pain [44]. It has even been suggested that the examination of a few selected points may be sufficient to identify FMS [45]. It is also argued that tender points can be faked, and that they truly bear no consequence to the composite of suffering of FMS.

Taking into account the presence of symptoms other than pain and the questions posed by tender points, new criteria for a diagnosis of FMS have recently been published [5]. These new criteria, which may be viewed as complementary to the 1990 criteria, with the elimination of the tender point examination, perform well in identification of patients with a previous diagnosis of FMS [5]. The new criteria therefore have included other symptom domains and made them an essential part of the criteria set. However, elimination of tender points also decreases specificity. Thus, unlike the 1990 ACR criteria, the new 2010 criteria require exclusion of other conditions causing pain. Further, these criteria have not been validated in primary care setting. A recent German working group has concluded that FMS not only can be diagnosed for clinical purposes on the basis of symptoms without a tender point examination, but may also be established using the old 1990 ACR criteria [46]. Therefore, wisdom suggests that a clinical diagnosis of FMS today should not be dependent solely upon a subjective count of tender points, but physicians may continue to use them for the present time in order to help solidify a diagnosis, as well as diagnosis of concomitant diseases, including depression [44, 47].

### 2.4. Fibromyalgia Is No Longer a Diagnosis of Exclusion

As early as 1989, Yunus has advocated that a diagnosis of FMS should not be made by exclusion, but rather by positive assessment of a constellation of symptoms [48]. Additionally, this clinical diagnosis should be made in the primary care setting without need for excessive and costly investigation or repeated specialist referral [16]. A detailed medical history and good physical examination can be used to exclude other rheumatologic conditions [8, 49].

Primary care physicians will be the first to evaluate and manage patients with a complaint of body pain due to FMS [16, 50, 51]. Family physicians are likely best suited for the care of these patients in view of the multiplicity of complaints and need for therapies that span many categories [16]. It is also questionable whether there is truly a need for the diagnosis to be confirmed by a medical specialist such as a rheumatologist. The value of specialist opinion should be for the patient in whom some other condition requires exclusion, rather than to confirm the diagnosis of FMS. 

The clinical presentation of FMS can however be quite diverse with some areas of the body more painful than others, fluctuations in intensity of pain and variable intensity of other associated symptoms. Patients also differ considerably in terms of severity of functional impairment [52, 53]. This awareness of differences in presentation and heterogeneity of FMS is increasingly appreciated and is helpful to the clinician. 

It is also time to dispel the fallacy that FMS is a primary psychogenic condition. As mentioned earlier, depression and FMS are biologically different conditions and depression is present in any chronic diseases, including those with organic pathology, such as cancer and coronary artery diseases. There is no question that the psyche and the body are tightly linked. Illness, particularly prolonged and poorly recognized, fosters mood disturbance. Conversely, mood disorder is associated with reduced motivation to be physically active, resulting in muscle deconditioning and subsequent pain complaint. FMS patients have a greater prevalence of lifetime as well as current mood disorder compared to other populations, but this should not be viewed as causative in each and every patient. It is possible that a vulnerable psychological status may predispose an individual to onset of a pain process, particularly if triggered by some event [54–56].

A clinical evaluation can be used to exclude most other conditions that could masquerade as FMS [49, 57]. Some common medical conditions that should not be missed in a patient presenting with chronic widespread pain include hypothyroidism, statin-induced myopathy, and polymyalgia rheumatica, especially in the older patient. These are familiar to primary care physicians and should not cause confusion. Of greater concern is that a diffuse pain syndrome may either herald or mask a treatable rheumatologic condition such as rheumatoid arthritis, systemic lupus erythematosis, or a neurological illness such as multiple sclerosis, although this has only rarely been recorded in a few prospective studies.

## 3. What Causes Fibromyalgia?

Although the exact cause of FMS is unknown, abnormalities of nervous system pain processing can plausibly explain the persistence of pain in the absence of tissue damage [58]. The reason for this dysregulation is likely dependent upon a number of interacting factors, which include genetic predisposition, neurophysiological changes, and abnormal stress response. As following articles in this publication will elaborate further on pathogenesis, we will provide only a brief overview.

### 3.1. Genetic Factors

The evidence for genetic predisposition stems from studies showing familial aggregation of FMS [59, 60]. The odds ratio for a diagnosis of FMS was reported as 8.5 for a patient with a first-degree relative with FMS compared to having a relative with rheumatoid arthritis [60]. Familial aggregation should be a clue for some genetic contribution, but does not concretely prove a genetic link, as factors such as environment or a triggering event need to be taken into consideration. Candidate genes implicated in FMS include those controlling serotonin mechanisms, dopamine receptors, as well as metabolism of catecholamines [7, 61].

### 3.2. A Vulnerable Psychosocial Setting

Psychologic and stress-related factors are the second area of consideration regarding pathogenesis of FMS. Up to 40% of patients' report the onset of symptoms preceded by some triggering event, which might be either psychological or physical [62]. An abnormal physiological response to a stress mechanism could explain this phenomenon [54–56]. Impairment of the stress response, as measured by hypothalamo-pituitary-adrenal (HPA) axis response, was identified prior to the onset of chronic widespread pain in a prospective population-based study in England [63]. Psychological factors therefore play an important role in neurophysiological responses and have even been shown to affect changes at the spinal level [64].

Although FMS patients have a greater lifetime frequency of depression [65], depression per se appears not to be a direct causative factor in the pathogenesis of FMS [66]. Psychological symptoms, of which depression and anxiety are the most common, are however present in between 30 and 80% of FMS patients and contribute to poor global health [66].

### 3.3. Neurophysiological Changes

The concept of a neurological versus a purely somatoform disorder to explain the pathogenesis of FMS continues to stimulate debate [1, 67]. There is however convincing documentation of changes at various levels of the nervous system supporting an abnormality that is primarily neurogenic and will be further elaborated in ensuing articles in this supplement. A recent development in the pathophysiology of FMS is that it is characterized by central sensitization (CS), well documented in the laboratory setting [20, 21, 68]. CS also binds FMS to other similar syndromes, for example, IBS and TMD, collectively known as central sensitivity syndromes [20, 21].

Chronic pain, as occurs in FMS, may be perpetuated by numerous interacting mechanisms, including increased excitation or reduced inhibition [58, 69]. Neurophysiologic studies have demonstrated evidence of dysfunctional central pain mechanisms including spinal hyperexcitability [68, 70], changes in thalamic and cortical pain matrix, as well a grey matter volume [71, 72], and impaired function of normal descending inhibitory mechanisms [73, 74]. In simple terms, there is evidence for excessive pain-related neuronal activity at multiple levels of the central nervous system, structural, and functional changes in the brain by imaging studies and impaired function of normal descending inhibitory mechanisms.

## 4. Treatment Challenges

Treatments will be fully addressed in another article of this supplement. We will however provide a brief introduction by emphasizing new concepts that apply to management of FMS. Firstly, the concept of symptom-based treatments is logical and will allow a focussed starting point for a physician. Secondly, in the setting of no single “gold standard” treatment, a multimodal approach which includes both nonpharmacologic and pharmacologic treatments is rational [75–77]. In this regard, patient education with emphasis on an active role of the patients is critical. A patient-centred approach with individualization of management is very important.

Finally, as patients with FMS commonly report sensitivity to medications, clinical experience suggests that low doses of medications can be used, with gradual increase in dose depending upon efficacy and tolerability. It is the authors' experience that doses of medications used in real life clinical practices are often much lower than those reported in industry-controlled studies. It is also notable that many of the adverse effects of medications present symptoms similar to those experienced by patients with FMS. Therefore, any patient being treated with a medication should be carefully evaluated for both efficacy as well as side effects, and medications should be discontinued unless there is evidence for definite benefit. In addition, combinations of medications are also more commonly used in practice, although there is limited evidence to support this practice from randomised clinical trials. 

A key principle to management of patients with FMS is to encourage a shift of locus of control towards the patient and to ensure that the patient is an active rather than a passive participant in management. Understanding and support should form the cornerstone of care for these patients, with treatment strategies directed towards psychological status and physical symptoms within the context of family and society. Most patients will eventually with time find some treatment modality which will at least somewhat modulate, but not cure symptoms, improve health status globally and improve function.

Evidence-based treatment guidelines include those developed by the American Pain Society (APS) in 2005 and the European League Against Rheumatism (EULAR) in 2008 [49, 78]. Recent reviews of treatment options state that there is good to moderate evidence for efficacy of over 20 treatment interventions in FMS, highlighting the uncertainty in management of these patients [79]. Approval of several drugs by the FDA in recent years, for example, pregabalin, duloxetine, and milnacipran, has been of great help in alleviating symptoms. Other medications may also be used [77].

Nonpharmacologic treatments are an important component of management and recommended in both sets of guidelines. These might include a tailored exercise program, water therapy, physiotherapy, relaxation, cognitive behavioural training, and psychological support [80].

## 5. Outcome Is Not Universally Bleak

Factors that can help predict the outcome for patients with FMS are as yet not fully understood, but patients that do well are more likely to be engaged in physical activity and use less medication. Realistic outcome goals should be emphasized. Reduction of symptoms should be translated into improved functional status. When a patient reports improvement in symptoms without a parallel functional change, the physician should question first the true efficacy of the treatment, secondly the side effect profile which might be contributing to poor effect on function, and finally patient motivation to achieve an improved health status.

A study of outcome done by postal questionnaire in the USA suggested continued pain and disability, with little change over time [52, 53]. However, publications from Australia, Mexico, and Canada have reported a more favourable outcome [81–83]. In a Canadian prospective study of FMS patients, almost 50% reported a clinically meaningful improvement in overall status of FMS over a 3-year observation period [83]. This improvement in outcome is further supported by the findings that 65% of subjects improved over a 2-year period in a community-based study in England [84]. Good long-term prospective studies are still lacking and many questions regarding prognosis and outcome remain unresolved.

## 6. Conclusion

There is accumulating and ample evidence in the scientific literature to support the existence of FM as an entity and as a diagnosable condition by its own characteristic features, even in the absence of an objective clinical test. There has been a substantial progress in an understanding of the pathophysiology of FMS in the past 20 years. Now it is known that it is a neurobiological disease. The mechanisms predominantly involve central sensitization, contributed by genetics, endocrine factors, poor sleep, psychosocial and physical stress, and physical trauma. 

The initial clinical challenge is to correctly diagnose FMS on the basis of patient report and in the absence of an objective clinical test to confirm diagnosis or gauge severity of symptoms. Therefore, the accurate recognition of FMS and assessment of response to treatments still require the time-honoured art of clinical medicine [85]. There is currently no cure for FMS, and no single treatment is universally effective for control of pain and the associated features of this condition. Even in the absence of complete understanding of cause and pathogenesis, treatments can be directed to alleviate symptoms with the goal to improve functional status. Global outcome with attention to overall well-being represents a more realistic outcome measure that is both clinically applicable and pertinent to the patient. The translation of the knowledge of the pathogenesis of FM has however greatly facilitated introduction of treatment options, including use of pharmacological agents, that are finally beginning to show promise for the management of this illness.
